# Psychometric properties of the Persian version of the childhood epilepsy questionnaire-16 (QOLCE-16) in a sample of parents of children with epilepsy

**DOI:** 10.1186/s12955-026-02504-0

**Published:** 2026-02-25

**Authors:** Hamid Nemati, Parvin Ghaemmaghami, Leila Roosta, Seyedeh Leila Dehghani, Marzieh Alamolhoda

**Affiliations:** 1 Shiraz Neuroscience Research Center, Shiraz University of Medical Sciences, Shiraz, Iran; 2 School of Nursing and Midwifery, Shiraz University of Medical Sciences, Shiraz, Iran; 3 Behbahan Faculty of Medical Sciences, Behbahan, Iran

**Keywords:** Epilepsy, Children, Health-related quality of life, QOLCE, Translation, Reliability, Validity

## Abstract

**Background:**

As a chronic neurological condition, epilepsy adversely affects multiple dimensions of quality of life in pediatric and adolescent populations. The aims of the study were translation and cultural adaptation (TCA) of the Persian version of the 16-item Quality of Life in Childhood Epilepsy (QOLCE-16) and assessment of its psychometric properties in children and adolescents with epilepsy.

**Methods:**

A total of 290 parents of children with epilepsy were eligible to participate in this study and completed the questionnaire. TCA procedures were used to adapt the English version of the QOLCE-16 into Persian. Reliability, internal consistency, item analysis, test-retest reliability, convergent validity, discriminant validity, and confirmatory factor analysis (CFA) were conducted to evaluate the psychometric properties of the Persian version of the QOLCE-16.

**Results:**

Internal consistency measured by Cronbach’s alpha coefficients was excellent, ranging from 0.80 to 0.92. The items were significantly related to the total scores (*p* < 0.05). Intraclass correlation coefficients were satisfactory across all domains. Convergent and discriminant validity were supported for all subscales based on predefined correlation-based criteria. The results of CFA confirmed a second-order model with excellent fit based on significant standardized estimates of factor loadings and favorable goodness-of-fit indices (CFI = 0.979, TLI = 0.973, RMSEA = 0.062, 90% CI [0.048, 0.076], and SRMR = 0.062).

**Conclusions:**

The Persian version of the QOLCE-16 has good reliability and validity in assessing the health-related quality of life of children with epilepsy in Iran. The translated version, as a brief and valuable measurement tool, can be applied in clinical research of a Persian-speaking population.

**Supplementary Information:**

The online version contains supplementary material available at 10.1186/s12955-026-02504-0.

## Background

Childhood epilepsy is among the most common neurological disorders, characterized by recurrent and unpredictable seizures caused primarily by abnormal electrical discharges in a set of brain neurons [1]. Epidemiological studies have shown that more than 60% of individuals experience their first seizure before the age of 18 [2]. Over 291 million children and adolescents under the age of 20 worldwide are affected by epilepsy and cognitive impairment [3]. With approximately 30,000 new cases annually, epilepsy is recognized as the most common chronic neurological disorder among children and adolescents globally [4]. Due to its widespread occurrence and related comorbidities, epilepsy has emerged as a significant public health challenge.

Pediatric studies on seizure disorders have indicated that children diagnosed with epilepsy experience significantly higher rates of psychiatric comorbidities, including anxiety, depression, attention-deficit hyperactivity disorder (ADHD), behavioral problems, and developmental delays, compared to healthy children [5, 6]. The presence of recurrent seizures and limitations in physical activities, psychological and social dysfunction, and adverse effects of the treatments significantly undermine the quality of life (QoL) in children living with epilepsy [7–12].

Health-related quality of life (HRQoL), a multidimensional construct, evaluates how health status influences physical, psychological, and social well-being. It is recognized as one of the most important health outcomes in chronic diseases [13]. Assessment of HRQoL provides suitable feedback on a patient’s overall health status and serves as an effective tool for evaluating the well-being of individuals with epilepsy [14]. Therefore, selecting a suitable HRQoL measure ensures that healthcare efforts are not solely focused on long-term care and reducing seizure frequency but also address the patient’s fundamental needs, their ability to adapt to the social environment, and their overall satisfaction with their health status [15].

Several instruments have been developed to assess the QoL in children with epilepsy (CWE) [16]. According to a systematic review of the measurement properties of epilepsy-specific Patient Reported Outcomes Measures (PROMs), Quality of Life in Childhood Epilepsy (QOLCE) questionnaires are more frequently used than other PROMs [16]. QOLCE is designed for parents of CWE, and its reliability and validity have been confirmed across countries and cultural settings [17–20]. The initial QOLCE was an epilepsy-specific measure that included 73 items with 16 subscales covering 7 domains: cognition, physical activities, social activities, emotional well-being, behavior, general health, and general quality of Life [21]. After that, a revised version with 76 items was validated in a sample of American epilepsy patients [20]. Due to the extended time required to complete the questionnaire, a modified version reduced the number of items to 55 with four main domains [22, 23]. Subsequently, in 2018, Goodwin et al. used an item response theory model to develop a brief 16-item version from the QOLCE-55, categorized into four domains: cognitive, emotional, social, and physical functioning, with four items in each of the subscales [14, 24]. Although concise, the QOLCE-16 retains good psychometric properties, mirroring the original instrument’s reliability in assessing HRQoL among CWE. The results of published studies using QOLCE-76 and QOLCE-55 were comparable to those conducted using the QOLCE-16 in terms of structural validity [16].

The English version of the QOLCE-16 has been translated and culturally adapted as a valid and reliable instrument into other languages and cultures [24–28]. To the best of our knowledge, a Persian version of the QOLCE-16 has not yet been developed or psychometrically evaluated. Given the cultural context, a Persian adaptation may serve as a suitable and valid tool for assessing HRQoL in Iranian CWE. The aims of the present study were the translation and cultural adaptation of the QOLCE-16 into Persian and the evaluation of the psychometric properties of the Persian version, including its reliability and validity, to assess its cultural compatibility and applicability within the Iranian CWE population.

## Methods

In the first stage of the study, the questionnaire was translated and culturally adapted for use in the Persian-speaking population. In the second stage, we evaluated the reliability, validity, and psychometric properties of the Persian version of the QOLCE-16. The translation and cultural adaptation process adhered to established guidelines for the adaptation of self-report measures [29, 30] and aligned with the principles of good practice for the translation and cultural adaptation of patient-reported outcome (PRO) measures [31] as outlined below.

### Translation and cultural adaptation

The forward-backward translation procedure was employed to maintain conceptual equivalence with the original questionnaire. Initially, permission was obtained from the original developers of QOLCE-16. The questionnaire was then independently translated into Persian by two bilingual translators whose native language was Persian. The two translations were reviewed, compared, and synthesized to produce a preliminary Persian version. Subsequently, the back-translation into English was conducted by two native English-speaking translators who were blinded to the original version and the purpose of the study.

The back-translations were reconciled into a unified English version through iterative review. This final English version was then carefully compared and analyzed against the original questionnaire. Following minor revisions, a panel of experts, including a neurologist and two bilingual English Persian translators, reached a consensus on the finalized Persian version of the QOLCE-16 for use in the study.

Cognitive interviews were conducted with a randomly selected subset of 10 parents to assess the clarity and cultural relevance of the items. After completing the Persian version of the QOLCE-16, parents were asked to provide feedback on the meaning and interpretation of the words and statements, particularly if they found any items confusing or difficult to understand. None of the participants reported any issues regarding ambiguity or comprehension of the questionnaire items.

### Participants and instrument

This cross-sectional study included 349 parents or caregivers of children and adolescents diagnosed with epilepsy, who were visited at the pediatric neurology unit at Emam Reza Clinic, affiliated with Shiraz University of Medical Sciences in Shiraz, Iran, between March and June 2025.

Inclusion criteria were children aged 4 to 18 years with a confirmed diagnosis of active epilepsy, and parents or caregivers who were willing to participate in the study. Children with severe comorbidities, significant disabilities or mobility limitations, and parents with cognitive or mental impairments were excluded from the study.

Parents were asked to complete the QOLCE-16 questionnaire and provide written informed consent. Demographic variables collected included: child’s gender, age, age at epilepsy onset, school grade level, duration of epilepsy, parents’ age, educational level, average monthly household income, epilepsy type, last seizure time, and use of antiepileptic drugs (AEDs). The Ethics Committee of Behbahan Faculty of Medical Sciences approved the study protocol.

The QOLCE-16 questionnaire, a brief version of the QOLCE-55, is a multidimensional instrument with established psychometric properties for assessing HRQoL in CWE [24]. The 16-item questionnaire includes four domains: cognitive functioning (4 items), emotional functioning (4 items), social functioning (4 items), and physical functioning (4 items). Participants responded to items using a 5-point Likert scale: 1 = Very often, 2 = Fairly often, 3 = Sometimes, 4 = Almost never, and 5 = Never. Responses were recoded and linearly transformed according to the scoring instructions [24]. All items were converted to a 0–100 scale, with higher scores indicating better HRQoL [22, 24]. Additional file 1 provides a detailed description of the Persian version of QOLCE-16 [see Additional file 1].

### Psychometric evaluation and statistical analysis

The reliability and validity of the QOLCE-16 questionnaire were evaluated using the Classical Test Theory (CTT) approach. To assess reliability, Cronbach’s alpha coefficient was calculated. Internal consistency for each domain of the questionnaire was determined by computing Cronbach’s alpha value within that domain. Cronbach’s α values exceeding 0.70 were deemed acceptable for scale reliability [32]. The percentages of respondents obtaining the lowest and highest possible scores indicate the presence of floor and ceiling effects, respectively. Floor or ceiling effects are considered potentially problematic if more than 15% of participants’ responses are clustered at the extremes (i.e., the lowest or highest options) [33]. Test-retest reliability was assessed by calculating the Intraclass Correlation Coefficient (ICC), which quantifies the ratio of between-subject variability to total variability, reflecting stability over time. ICC values between 0.60 and 0.80 indicate good reliability, while values above 0.80 indicate excellent reliability [34]. To evaluate the differentiating ability of the questionnaire items, the critical ratio (CR) was calculated for each item. The CR values indicate how well each item differentiates between individuals with high and low total scores.

CR values for every item were calculated using independent-samples T-tests comparing the highest 27% and lowest 27% of respondents based on their total scores for each item. Values greater than 3 were considered statistically significant [35]. Additionally, Pearson’s correlation coefficients between each item and the total score were computed; significant correlations were interpreted as evidence of good item-total correlation [35].

To examine convergent validity, the Spearman correlation coefficients were calculated between each item and its corresponding domain scale. Correlation values above 0.40 indicate acceptable convergent validity. When the Spearman correlation between an item and its hypothesized domain is significantly higher than its correlations with other domains, discriminant validity is established [36]. Results of convergent and discriminant validity assessments were reported using the scaling success rate, which represents the percentage of items that correlate more strongly with their intended domain than with other domains.

The construct validity was evaluated using confirmatory factor analysis (CFA). As the QOLCE-16 is a previously developed instrument with a theoretically specified factor structure, CFA was employed to test whether the observed data fit the originally proposed measurement model in the Persian version of the questionnaire. CFA allows for hypothesis-driven evaluation of the relationships between observed items and their underlying latent constructs [37, 38]. In this study, a second-order CFA model previously proposed by Goodwin et al. was used, in which four first-order factors, cognitive, emotional, social, and physical functioning, loaded onto a second-order factor representing overall HRQoL [24].

Given the ordinal nature of the item responses and the violation of the multivariate normality assumption, parameters were estimated using the weighted least squares mean and variance adjusted (WLSMV) estimator [38, 39]. Model fit was evaluated using multiple goodness-of-fit indices, including the chi-square statistic (χ²), the Comparative Fit Index (CFI), the Tucker–Lewis Index (TLI), the Root Mean Square Error of Approximation (RMSEA) with its 90% confidence interval, and the Standardized Root Mean Square Residual (SRMR). Values of CFI and TLI ≥ 0.90 were considered indicative of acceptable fit, with values ≥ 0.95 reflecting excellent fit. RMSEA values ≤ 0.05 indicated close fit, values between 0.05 and 0.08 acceptable fit, values between 0.08 and 0.10 mediocre fit, and values > 0.10 poor fit. SRMR values < 0.08 were considered acceptable. These criteria were based on commonly recommended guidelines in the structural equation modeling literature [37].

Descriptive statistics and psychometric analyses were conducted using SPSS (IBM SPSS Statistics, Armonk, NY, USA). CFA was performed using the lavaan package in R (version 4.5.2).

## Results

### Participant characteristics

Among the 349 parents of CWE initially recruited for the study, 59 were excluded due to not meeting the age criteria or because of cognitive impairments and comorbidities. Ultimately, 290 participants were included in the analysis. The mean age of the children was 10 years, with an average age of epilepsy onset at 6 years. Of the patients, 166 (57%) were boys. The mean duration of epilepsy was approximately 4 years. More than 44% of the children were in elementary school. 54% of the children suffered from Idiopathic Focal Epilepsy (IFE), and 147 (50.7%) were taking either no medication or only one antiepileptic drug. About 60% of the children experienced their last seizure more than six months ago.

Among respondents, 210 (73%) were mothers, with a mean age of 40 years. Nearly 60% had a diploma or higher education level, and most lived in households with an average income. Detailed demographic and clinical characteristics of the patients and their parents are presented in Table 1.

**Table 1:** Demographic and clinical characteristics of children with epilepsy and their parents/caregivers (*n* = 290)

Children		Parents/ caregivers	
Gender *n*(%)		Child’s relatives *n*(%)	
Boy	166 (57.20)	Father	68 (23.6)
Girl	124 (42.80)	Mother	210 (72.9)
**Mean age in years** (SD)	10.89 (3.80)	Grandparents	3 (1.0)
**Mean age of epilepsy onset in years** (SD)	6.65 (4.27)	Others	7 (2.4)
**Mean duration of epilepsy in years (SD)**	4.30 (3.91)	**Mean age (year)** (SD)	40.55 (6.79)
**Education** n(%)		**Education level** n(%)	
Haven’t started school	21 (7.4)	Illiterate	3 (1.1)
Kindergarten	21 (7.4)	Primary education	44 (15.4)
Primary school	126 (44.2)	Secondary education	55 (19.3)
Second school	45 (15.8)	Diploma	105 (36.8)
High school	23 (8.1)	University	78 (27.4)
Special school	16 (5.6)	**Income level** n(%)	
Others	33 (11.7)	Low	96 (33.4)
**Epilepsy types** n(%)		Medium	183 (63.7)
Idiopathic focal epilepsy (IFE)	148 (54)	High	8 (2.8)
Symptomatic focal epilepsy (SFE)	62 (22.6)		
Idiopathic generalized epilepsy (IGE)	51 (18.6)		
Symptomatic generalized epilepsy (SGE)	13 (4.7)		
**Last Seizure time (months) n(%)**			
**< 1**	24 (9.2)		
**1–6**	81 (31.0)		
**> 6**	156 (59.8)		
**Consumption of Anti-epileptic drugs n(%)**			
**0–1**	147 (50.7)		
**2–3**	133 (45.9)		
**> 3**	10 (3.4)		

### Reliability and agreement analysis

The Cronbach’s α was 0.91 for the overall scale. Table 2 presents the mean scores for each domain of the QOLCE-16, as well as the floor and ceiling effects and Cronbach’s α coefficients for four subscales. The mean values of the subscales ranged from 77.16 (SD = 28.10) in cognitive functioning to 81.23 (SD = 25.91) in social functioning. The floor and ceiling effects for the overall measure of QOLCE-16 were 1.1% and 11.5%, respectively. While no floor effects emerged, substantial ceiling effects (24.2–44.4%) were present across domains, peaking in social functioning.

**Table 2:** Internal consistency, test-retest reliability, convergent and discriminant validity for QOLCE-16 subscales (Persian version)

	Items	Mean (SD)	Floor effect(%)	Ceiling effect(%)	Cronbach’α	ICC	Convergent validity^*^		Discriminant validity^**^	
							Correlation rang	Scaling success (%)	Correlation rang	Scaling success (%)
Cognitive	4	77.16(28.10)	4.1	35.9	0.92	0.69	0.71–0.89	4/4 (100)	0.26–0.60	12/12(100)
Emotion	4	78.35(23.23)	0.8	24.2	0.80	0.77	0.69–0.83	4/4 (100)	0.26–0.52	12/12(100)
Social	4	81.23(25.91)	2.5	44.4	0.86	0.70	0.73–0.87	4/4 (100)	0.34–0.59	12/12(100)
Physical	4	77.64(27.37)	4.5	32.8	0.87	0.72	076-0.83	4/4 (100)	0.35–0.63	12/12(100)
Total	16	79.16(21.68)	1.1	11.5	0.91	0.83	-	-	-	-

SD Standard deviation, ICC Intraclass correlation coefficient

^*^Number of correlations between each item and its corresponding scale corrected for overlap ≥ 0.4/ total number of convergent validity tests

^**^Number of convergent correlations significantly higher than discriminant correlations/Total number of correlations

Internal consistency coefficients for the domains ranged from 0.80 to 0.92. In the test-retest reliability analysis, the ICC for 36 participants who completed the retest indicated acceptable reliability across all domains (ranged from 0.69 to 0.77) and the total score (ICC = 0.83, CI: [0.67–0.91]). Results from the item analysis presented in Table 3 showed that CR values for all items were above 3, and p-values for all items were statistically significant. Correlation coefficients between each item and the total score provided evidence of strong item-total correlations.

**Table 3:** Item analysis and factor loadings of exploratory factor analysis for QOLCE-16 subscales (*n* = 290)

Items	Mean (SD)	CR^*^	Item-total correlation^**^
Q1_cognitive	76.55(31.71)	14.52	0.75
Q2_cognitive	73.71(34.12)	14.27	0.77
Q3_cognitive	87.59(26.41)	7.59	0.72
Q4_cognitive	70.77(32.91)	14.09	0.73
Q1_emotion	76.74(30.51)	9.55	0.57
Q2_ emotion	75.57(32.01)	11.63	0.63
Q3_ emotion	86.25(28.06)	7.63	0.62
Q4_ emotion	74.36(25.67)	11.51	0.67
Q1_ social	82.24(31.46)	9.94	0.76
Q2_ social	85.85(24.60)	9.52	0.69
Q3_ social	83.62(27.69)	11.37	0.76
Q4_ social	76.28(31.26)	14.68	0.80
Q1_physical	76.47(34.05)	13.20	0.75
Q2_ physical	82.41(28.46)	10.74	0.75
Q3_ physical	84.52(28.57)	8.65	0.67
Q4_ physical	67.57(35.41)	16.28	0.73

^*^ CR Critical ratio ^**^ All p-values are significant at 0.05 level

### Validity analyses

The results of convergent validity are presented in Table 2, showing the highest correlations between each item and its respective subscale score. Additionally, the scaling success rates were 100% across all domains of the questionnaire, indicating excellent discrimination between items within a domain and items from other domains.

**Fig. 1:**
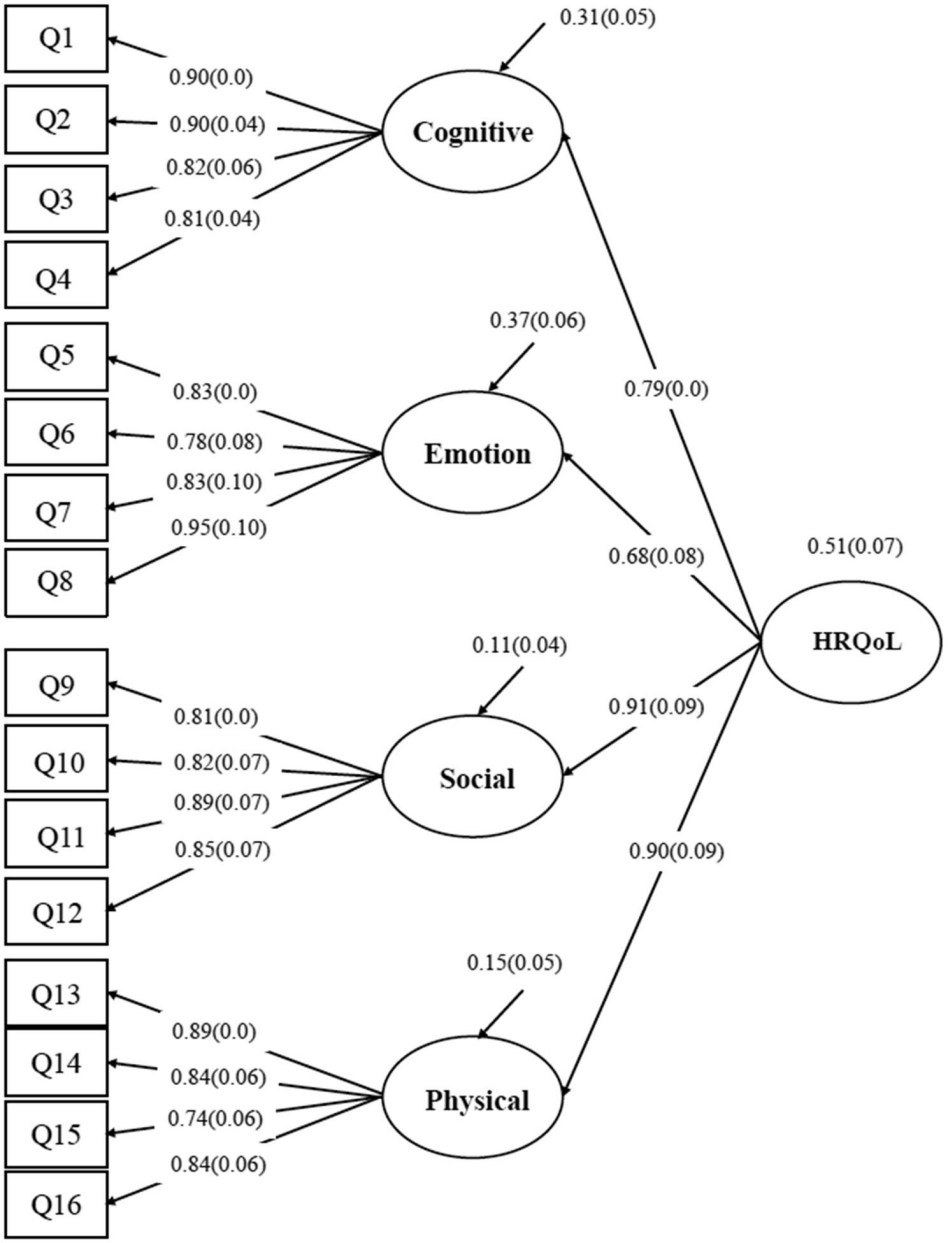
Path diagram for the second-order confirmatory factor model of Persian version of the QOLCE-16. All standardized estimates of the factor loadings were statistically significant at *p* < 0.05 level

The initial second-order CFA model demonstrated acceptable but suboptimal fit (χ² = 279.86, df = 100, *p* < 0.001; CFI = 0.955, TLI = 0.946, RMSEA = 0.088 [90% CI: 0.076–0.101], SRMR = 0.082). Based on modification indices and theoretical considerations, residual correlations were introduced between items within the same dimensions, namely emotional, social, and physical, with no changes to the overall factor structure. The modified CFA model showed improved fit (χ² = 178.17, df = 94, *p* < 0.001; CFI = 0.979, TLI = 0.973, RMSEA = 0.062 [90% CI: 0.048–0.076], SRMR = 0.062), with all standardized factor loadings remaining strong and statistically significant. These results support the robustness of the second-order factor structure of the Persian QOLCE-16. Fig. 1 presents the standardized factor loadings and their standard errors.

## Discussion

The aim of the study was the translation and cultural adaptation of the English version of QOLCE-16 into Persian. The study attempted to evaluate the psychometric properties (including reliability, internal consistency, test-retest reliability, and construct validity) of the Persian version of the QOLCE-16 in Iranian CWE using Classical Test Theory (CTT). Psychometric analyses confirmed the Persian QOLCE-16 as a robust measure of QoL. Therefore, it can serve as an appropriate instrument for evaluating HRQoL in Iranian epilepsy children.

High values of QoL scores across all domains indicated that the children in this study experienced relatively good QoL. The results of the item analysis were satisfactory. All items showed significant CR and strong item-total correlations, demonstrating satisfactory reliability. These findings are consistent with the findings of a Chinese QoL study, supporting the suitability of the Persian version of the QOLCE-16 for measuring variability in QoL among CWE [28].

Floor effects ranged from 0.8 to 4.5% and remained within acceptable limits. However, high ceiling effects were found in the QOLCE-16, ranging from 24.2 to 44.4%, with the highest value in social functioning. No floor effects and notable ceiling effects in all subscales of the QOLCE-16 mean that most parents have not been able to differentiate between “sometimes” or “fairly often” and “very often” options in meaning. Moreover, the high scores ceiling effect on the social functioning indicated social interactions in CWE and peer acceptance similar to QoL studies of children with heart disease and cerebral palsy [40–42]. Children in this study experienced a low seizure frequency per month, with most of them not taking any or one anti-epileptic drug. A considerable proportion of children do not have severe conditions that would be expected to influence their QoL scores.

The Cronbach’s alpha coefficient for the Persian version was high (0.91), like the original English version of the QOLCE-16 and other language versions [24, 26–28]. The internal consistency coefficients for all domains of the Persian version exceeded 0.80, indicating strong homogeneity and inter-dependence, which means that each domain measures a specific concept or construct [43]. Test-retest reliability demonstrated acceptable-to-good stability, aligning with prior validation studies of both the 16-item and longer QOLCE versions [24, 26, 28, 44–46]. Convergent and discriminant validity results were excellent. High correlation ranges and 100% scaling success rates across all subscales indicated strong convergent validity between each item and its intended subscale. Significant inter-domain correlations also confirmed the questionnaire’s ability to differentiate well between domains.

CFA confirmed the proposed second-order model, demonstrating that the four primary domains, cognitive, emotional, social, and physical functioning, converged to form a higher-order factor representing overall HRQoL. The standardized factor loadings ranged from 0.68 to 0.91 and were statistically significant (p-value < 0.05). Although the chi-square statistic remained statistically significant, this is a well-known consequence of its sensitivity to sample size and minor deviations from model assumptions [47]. Other fit indices, including CFI, TLI, RMSEA, and SRMR, indicated excellent model fit. CFA results showed HRQoL is a multidimensional construct encompassing cognitive, emotional, social, and physical factors, each measured by four items, which are consistent with the original QOLCE-55 and QOLCE-16 versions, as well as other validation studies [22, 24, 26, 27].

As the Persian version of the QOLCE-16 was applied for the first time in Iranian CWE, exploratory factor analysis (EFA) was conducted prior to CFA as a preliminary evaluation to examine whether the underlying factor structure of the translated instrument was preserved in the new linguistic and cultural context. The results indicated that the data were suitable for factor analysis, and the extracted subscales explained a substantial proportion of the total variance (69.9%). Most items loaded strongly on their intended domains, supporting the preservation of the original four-factor structure. Two items, “felt confident?” (emotional subscale) and “needed more supervision than other children his/her age?” (physical subscale) showed stronger loadings on domains other than those hypothesized which likely reflects cultural or linguistic nuances rather than structural deficiencies. Importantly, in the CFA of the Persian version, both items remained within their original domains. In cultural adaptation studies, such variations underscore the importance of carefully considering item interpretation within different populations. Similar findings have been reported in studies on QoL among Iranian children with diabetes and ADHD [48, 49].

The brief version of QOLCE-16, developed by Goodwin et al., provides evidence of good measurement properties, conforms to the factor structure of the QOLCE-55, reduces completion time, and is a suitable tool for use in assessing HRQoL in CWE. Using the short version allows researchers to measure HRQoL in CWE accurately and quickly in clinical research [14]. Our analyses confirm that the Persian adaptation maintains the psychometric integrity of the original instrument for HRQoL assessment in pediatric epilepsy. It successfully captured all the dimensions included in the original version and replicated previously published findings [26–28]. As demonstrated by Goodwin et al., this short form also shows measurement equivalence across gender, age, and time groups in the original validation study [14]. Measurement invariance across cultural groups was not evaluated in the present study. Future research should examine measurement invariance between the Persian and Canadian versions of the questionnaire to ensure the validity of cross-cultural comparisons.

This study had three main limitations. First, the cross-sectional study was conducted only in a neurological clinic in southern Iran. Therefore, generalization of the results should be made to all patients with caution. Second, although convergent and discriminant validity were supported by Spearman correlation coefficients among scale items and domains, the QOLCE-16 was not compared with other measures to establish validity. Future studies need to be carried out on this questionnaire with other validated measures designed for children and adolescents. Finally, due to potential clinical changes over time, a longitudinal study is warranted to evaluate the questionnaire’s power to detect changes in QoL over longitudinal intervals.

## Conclusions

The results of this study demonstrate that the Persian version of the QOLCE-16 is a reliable and valid instrument for assessing HRQoL in Iranian children and adolescents with epilepsy. Furthermore, the findings confirm that the 16-item Persian version retains the good psychometric properties of its original version. Its brevity, ease of use, and time efficiency may increase the accuracy of responses and reduce respondent burden, making it a practical alternative to longer epilepsy-related QoL questionnaires for pediatric populations. Therefore, the Persian QOLCE-16 can be recommended for both clinical and research purposes in Iran.

## Supplementary Information

Below is the link to the electronic supplementary material.

Supplementary Material 1

Supplementary Material 2

## Data Availability

The datasets used or analyzed during the current study are available from the corresponding author on reasonable request.

## Abbreviations

QOLCE-16: Childhood Epilepsy Questionnaire-16
TCA: Translation and cultural adaptation
ADHD: Attention-Deficit Hyperactivity Disorder
CWE: Children with Epilepsy
QoL: Quality of Life
HRQoL: Health Related Quality of Life
CSP: Child Seizure Profile
PRO: Patient-reported outcome
CTT: Classical Test Theory
ICC: Intraclass Correlation Coefficient
CR: Critical Ratio
CFA: Confirmatory Factor Analysis
WLSMV: Weighted least squares mean and variance adjusted
CFI: Comparative Fit Index
AGFI: Adjusted Goodness of Fit Index
TLI: Tucker–Lewis index
SRMR: Standardized Root Mean Square Residual
RMSEA: Root Mean Square Error of Approximation
IFE: Idiopathic Focal Epilepsy
