# Supplementary material for: Psychometric properties of the Persian version of the childhood epilepsy questionnaire-16 (QOLCE-16) in a sample of parents of children with epilepsy
Source: Health Qual Life Outcomes. 2026 Feb 25;24:46. doi: 10.1186/s12955-026-02504-0 (PMC13040998; doi:10.1186/s12955-026-02504-0)
Supplement: Supplementary file 1 — Supplementary Material 1 [file 12955_2026_2504_MOESM1_ESM.pdf]

پرسشنامه کیفیت زندگی در کودکان مبتلا به صرع

QOLCE-16 Version 1.0 (English)

سوالات زیر در مورد وضعیت سلامت و رفاه فرزند شما می باشد. لطفا گزینه مناسب را در هر سوال علامت بزنید. برخی از سوالات ممکن است شبیه به هم به نظر برسند اما با هم متفاوت هستند. برخی از سوالات ممکن است در مورد مشکلاتی باشد که فرزند شما ندارد. خواهشمند است به همه سوالات پاسخ دهید، چرا که دانستن اینکه فرزند شما این مشکلات را ندارد نیز برای ما مهم است. هیچ پاسخ درست و یا غلطی وجود ندارد. اگر در پاسخ به سؤالی مطمئن نیستید، بهترین پاسخی را که می‌توانید، انتخاب کنید.

بخش اول: عملکرد شناختی فرزند شما

سوالات زیر در مورد برخی از مشکلاتی است که کودکان در تمرکز، به خاطر سپردن و صحبت کردن دارند

در مقایسه با سایر کودکان هم سن، فرزند شما چه مدت در ۴ هفته گذشته:

	خیلی اوقات	نسبتا اغلب اوقات	بعضی اوقات	تقریبا هیچ وقت	هیچ وقت	غیر قابل اجرا
الف) در درک جهت ها و مسیرها مشکل داشته است؟	☐	☐	☐	☐	☐	☐
ب) در دنبال‌کردن دستورالعمل‌های پیچیده مشکل داشته است؟	☐	☐	☐	☐	☐	☐
پ) در دنبال‌کردن دستورالعمل‌های ساده مشکل داشته است؟	☐	☐	☐	☐	☐	☐
ت) در بخاطر سپردن چیزهایی که دیگران به او گفته اند مشکل داشته است؟	☐	☐	☐	☐	☐	☐

بخش دوم: عملکرد عاطفی فرزند شما

سوالات زیر به طور کلی احساس کودک شما را توصیف می کند.

فکر می کنید فرزند شما در ۴ هفته گذشته چه مدت:

	همیشه	اغلب اوقات	برخی اوقات	کمی از اوقات	هیچ وقت	غیر قابل اجرا
الف) احساس کرده که هیچ‌کس او را درک نمی‌کند؟	☐	☐	☐	☐	☐	☐
ب) احساس ناراحتی یا افسردگی داشته است؟	☐	☐	☐	☐	☐	☐
پ) احساس نا امیدی کرده است؟	☐	☐	☐	☐	☐	☐
ت) احساس کرده که اعتماد به نفس دارد؟	☐	☐	☐	☐	☐	☐

بخش سوم: عملکرد اجتماعی فرزند شما

سوالات زیر برخی از تعاملات و فعالیت های اجتماعی کودکان را توصیف می کند.

الف) در ۴ هفته گذشته، به دلیل صرع یا مشکلات مربوط به صرع، فعالیت های اجتماعی فرزند شما در مقایسه با سایر هم سالانش چقدر محدود بوده است؟

بله، خیلی محدود بوده است ☐

بله، تا حدی محدود بوده است ☐

بله، کمی محدود بوده است ☐

بله، به ندرت محدود بوده است ☐

خیر، محدود نبوده است ☐

غیر قابل اجرا ☐

در ۴ هفته گذشته، بیماری فرزند شما چه مدت:

	خیلی اوقات	نسبتا اغلب اوقات	بعضی اوقات	تقریبا هیچ وقت	هیچ وقت	غیر قابل اجرا
ب) بر تعاملات اجتماعی او و در مدرسه یا کار تأثیر گذاشته است؟	☐	☐	☐	☐	☐	☐
پ) باعث انزوای او از دیگران شده است؟	☐	☐	☐	☐	☐	☐
ت) حفظ دوستان را برای او دشوار کرده است؟	☐	☐	☐	☐	☐	☐

بخش چهارم: عملکرد جسمی فرزند شما

سوالات زیر در مورد فعالیت های بدنی است که ممکن است فرزند شما انجام دهد.

در ۴ هفته گذشته، فرزند شما در انجام فعالیت‌های روزانه‌اش ، چه مدت:

	خیلی اوقات	نسبتا اغلب اوقات	بعضی اوقات	تقریبا هیچ وقت	هیچ وقت	غیر قابل اجرا
الف) مانند سایر کودکان هم‌سن و سالش به‌راحتی در بیرون از خانه بازی کرده است؟	☐	☐	☐	☐	☐	☐
ب) توانسته است فعالیت‌های بدنی مشابه سایر کودکان هم‌سن خود انجام دهد؟	☐	☐	☐	☐	☐	☐
پ) همانند بچه های هم سن و سالش به راحتی در خانه بازی کرده است؟	☐	☐	☐	☐	☐	☐
ت) نیاز به نظارت بیشتری نسبت به سایر کودکان هم سن خود داشته است؟	☐	☐	☐	☐	☐	☐
