# Supplementary material for: Psychometric properties of the Persian version of the childhood epilepsy questionnaire-16 (QOLCE-16) in a sample of parents of children with epilepsy
Source: Health Qual Life Outcomes. 2026 Feb 25;24:46. doi: 10.1186/s12955-026-02504-0 (PMC13040998; doi:10.1186/s12955-026-02504-0)
Supplement: Supplementary file 2 — Supplementary Material 2 [file 12955_2026_2504_MOESM2_ESM.pdf]

Quality of Life in Childhood Epilepsy Questionnaire: QOLCE-16

Version 1.0 (English)

The following questions are about the health and well-being of your child. Please select the most appropriate response for each question. Some questions may seem similar but are different. Some questions may address issues that your child does not have. However, we ask that you respond to all questions because knowing that your child does not have these issues is important for us. There are no rights or wrong answers. If you are unsure how to answer a question, choose the best answer you can.

Section 1: Your Child's Cognitive Functioning

The following questions are about some difficulties that children may have with concentrating, remembering, and speaking. Compared to other children of the same age, how often has your child in the past four weeks:

	Very Often	Fairly Often	Sometimes	Almost Never	Never	Not Applicable
a) had trouble understanding directions and pathways?	☐	☐	☐	☐	☐	☐
b) had trouble following complex instructions?	☐	☐	☐	☐	☐	☐
c) had trouble following simple instructions?	☐	☐	☐	☐	☐	☐
d) had trouble remembering things that others have told him/her?	☐	☐	☐	☐	☐	☐

Section 2: Your Child's Emotional Functioning

The following questions generally describe how your child feels. In the past four weeks, how often do you think your child:

	Always	Often	Sometimes	Rarely	Never	Not Applicable
a) felt that no one understood him/her?	☐	☐	☐	☐	☐	☐
b) felt sad or depressed?	☐	☐	☐	☐	☐	☐
c) felt hopeless?	☐	☐	☐	☐	☐	☐
d) felt confident?	☐	☐	☐	☐	☐	☐

Section 3: Your Child's Social Functioning

The following questions describe some of the social interactions and activities of children.

a) In the past 4 weeks, how much have your child's social activities been limited compared to other children of the same age due to epilepsy or related issues?

Yes, very limited	☐
Yes, somewhat limited	☐
Yes, slightly limited	☐
Yes, rarely limited	☐
No, not limited	☐
Not Applicable	☐

In the past four weeks, how often has your child's condition:

	Very Often	Quite Often	Sometimes	Almost Never	Never	Not Applicable
b) affected his/her social interactions at school or work?	☐	☐	☐	☐	☐	☐
c) led to isolation from others?	☐	☐	☐	☐	☐	☐
d) made it difficult to maintain friendships?	☐	☐	☐	☐	☐	☐

Section 4: Your Child's Physical Functioning

The following questions are about physical activities that your child may engage in. In the past four weeks, how often has your child been able to:

	Very Often	Quite Often	Sometimes	Almost Never	Never	Not Applicable
a) play outside freely like other children of the same age?	☐	☐	☐	☐	☐	☐
b) do physical activities similar to other children of the same age?	☐	☐	☐	☐	☐	☐
c) play at home freely like other children of the same age?	☐	☐	☐	☐	☐	☐
d) required more supervision than other children of the same age?	☐	☐	☐	☐	☐	☐
